# Selenium reduces nociceptive response in acute 1-methyl-4-phenyl-1, 2, 3, 6-tetrahydropyridine (MPTP)-induced neurotoxicity

**DOI:** 10.1016/j.ibneur.2021.11.001

**Published:** 2021-11-27

**Authors:** Patrick Oluwole Abolarin, Abdulrazaq Bidemi Nafiu, Aboyeji Lukuman Oyewole, Abdulbasit Amin, Olalekan Michael Ogundele, Bamidele Victor Owoyele

**Affiliations:** a Department of Physiology, University of Ilorin, P.M.B 1515, Ilorin, Nigeria; b Department of Human Physiology, Federal University Dutse, Dutse, Jigawa State, Nigeria; c Department of Comparative Biomedical Sciences, Louisiana State University, School of Veterinary Medicine, Baton Rouge, LA, USA

**Keywords:** Neurotoxicity, Selenium, Motor coordination, Nociceptive threshold, Neuroinflammation, Pain biomarkers

## Abstract

The potential of Se to alleviate pain associated with 1-methyl-4-phenyl-1,2,3,6-tetrahydropyridine (MPTP)-induced neurotoxicity was investigated.

Swiss mice were *intraperitoneally* injected with MPTP (20 mg/kg) 4 times with an interval of 2 h in 1 day. Seven days after MPTP injection, the mice (n = 5 mice per group) were randomly assigned to groups: MPTP-, DOPA (50 mg/kg)-, Se4 (0.4 mg/kg)-, Se6 (0.6 mg/kg)-, DOPA+Se4-, and DOPA+Se6-treated groups were compared with controls. MPTP mice were treated for seven days; thereafter, motor-coordination and nociceptive-motor reactions were assessed. Pro-inflammatory cytokines (IL-1β, IL-6 and TNFα), and selected pain biomarkers (substance P (SP), glutamate and β-endorphin) were assessed in the serum and the substantial nigra pars compacta (SNpc).

Motor activity was increased slightly by Se (0.6 or 0.4 mg/kg) *vs*. MPTP (10.48 ± 2.71 or 11.81 ± 1.28 s *vs*. 3.53 ± 0.06 s respectively) but considerably increased by DOPA (50 mg/kg) *vs*. MPTP (50.47 ± 3.06 s *vs*. 3.53 ± 0.06 s respectively). Se and DOPA increased nociceptive threshold but Se alone reduced both serum and SN pro-inflammatory cytokines. Se modulates SP while DOPA modulates SP and glutamate in the SNpc of mice treated with MPTP.

Se suppressed pro-inflammatory cytokines and restored the basal pain biomarkers in the SNpc of mice treated with MPTP. Se requires further study as analgesic adjuvant.

## Introduction

Neurotoxicity due to 1-methyl-4-phenyl-1,2,3,6-tetrahydropyridine (MPTP) exposure has been used to model and study Parkinson’s disease (PD) in non-human primates and small rodents. PD is the second most common neurodegenerative disorder affecting 31–201 persons per 100,000 of aged populations worldwide. In Africa, this number has risen to about 436/100,000 of the aged population (Taba and Asser, 2004). The key feature of PD is the degeneration of dopaminergic (DA) neurons within the substantia nigra pars compacta (SNpc) leading to motor function deficits such as rigidity, bradykinesia, tremor, and postural instability (Jankovic, 2008). PD progression manifests with various non-motor symptoms such as pain, depression, sleep disorders and fatigue (Beiske et al. 2009).

About 40–60% of patients with PD experience various types of pain (acute or chronic) (Chaudhuri et al. 2006). Studies have shown that PD-associated pain is usually nociceptive (*i.e*. relating to tissue damage). Although, neuropathic pain (depicting pain that arises from nerve injury) and mixed pain syndrome (comprising both nociceptive and neuropathic pain) are also experienced by some PD patients. Pain may arise primarily from persistent tremor, muscle rigidity, dystonia, musculoskeletal injury, and inflammation (Snider et al., 1976, Quinn, 1999, Millan, 1999). Therefore, the management of PD-associated pain can be selected based on the type of pain (Wasner, Deuschl, 2012).

Management options for PD-associated pain include both pharmacological such as DA medications and non-pharmacological approaches such as physical therapy and exercise. Studies have shown that DA medications such as levodopa and benserazide combination (DOPA) reduce pain perception in people suffering from PD. Nonetheless, dyskinesia (which generates pain) often results from the prolonged usage of DOPA (Skogar and Lokk, 2016). Exercise is prescribed as a non-pharmacological management approach to PD-associated pain, however, poor exercise adherence makes physical therapy inefficient. Another underexplored option is the use of mineral micronutrients which are essential for normal growth and well-being. They can be used alone or in combination with other drugs as analgesic adjuvants.

Previous reports on the effects of mineral micronutrients on nociception and their potential use as analgesic adjuvants yielded varying results. *Intrathecal* Zn in mice produces antinociceptive effects especially in neuropathic pain. This effect was not significant in the thermoalgesia tests. Mg was also shown to inhibit mechanical hyperalgesia and induced antinociceptive effect in experimental neuropathic pain and clinical conditions. Felsby et al. (1996) recorded an ineffectiveness of Mg for peripheral neuropathy in a clinical study. Cu deficiency decreases brain level of dopamine in rats (Yu et al. 2008) and its preparations proved to be efficient monotherapy for pain management or analgesic adjuvant in combination with morphine. Tamba et al. (2013) observed a direct antinociceptive effect of Zn, Mg, and Cu. Selenium (Se), a widely reported mineral micronutrient with remarkable antioxidant and anti-inflammatory properties can potentially increase the pain threshold in MPTP-induced neurotoxicity.

Insufficient Se intake results in adverse health conditions like poor immune function and deterioration of cognitive functions (Gan et al., 2002, Balogh et al., 2004). A previous study has shown that Se deficiency potentiates neurotoxicity of methamphetamine in mice fed with a Se-deficient diet which leads to reduction of dopamine and its metabolites: 3,4-dihydroxyphenylacetic acid and homovanillic acid in the nigrostriatal tract (Kim et al. 1999). Se supplementation significantly blocked DA toxicity and protected neurons in the SNpc from damage caused by oxidative stress (Imam et al., 1999, Zafar et al., 2003). Neurotoxicity, in the MPTP-induced PD model, was aggravated by a Se-deficient diet and was improved by a Se-supplemented diet in mice (Kim et al. 2000). Se is a cofactor of glutathione peroxidase (GPx), a scavenging enzyme that plays a central role in removing excess H_2_O_2_ in the brain tissue (Rotruck et al., 1973, Li et al., 1990). Accumulated evidence correlates GPx activity and resistance to oxidative damage to level of Se intake (Castaño et al., 1993, Huang et al., 1994, Jiménez‐Jiménez et al., 1995).

The neurotoxin, MPTP, was discovered in the synthetic heroin, a drug of abuse that causes neurological symptoms similar to PD symptom (Langton, 2017). Perhaps the natural manifestations of PD may be due to chronic exposure to non-eco-friendly chemical analogs of MPTP. The chemical analogs that have been investigated for their toxicity includes 4′-fluoro-, 4′-chloro-, 2′-methyl- and 4′-amino-MPTP. Accidental exposure during manufacturing or clinical administration of desmethylprodine, an opioid drug and exposure to toxic industrial products such as pesticides are commonly reported source of exposure to MPTP chemical analogs. Park et al. (2015) reported that MPTP reduces the nociceptive threshold in mechanical and thermal nociception. Meanwhile, there has been no report on whether MPTP-induced neurotoxicity has effect on chemically induced nociceptive response.

Reports have shown that IL-1β is up-regulated in peripheral nerve and glial cells of the central nervous system following injury and trauma, respectively. This induces inflammatory hyperalgesia by increasing the synthesis of substance P (SP) and Prostaglandin E2 in glial and other neuronal cells (Zhang and An, 2007). On the other hand, IL-6 and TNFα were shown to enhance sensitivity to inflammatory and neuropathic hyperalgesia (Sweitzer et al., 2002, Schäfers et al., 2003). IL-1β mediates upregulation of SP in the SNpc (Thornton et al., 2010, Yang et al., 2015), decreases nociceptive threshold, augments blood-brain barrier permeability and induces microglial and astrocytes activation. Astrocytes activation potentiates synthesis of MAOB in the SNpc which increases the production of MPP+, the toxic metabolite of MPTP. This in turn increases the production of reactive oxygen species (ROS) that directly causes DA cell death in MPTP-induced PD model. Therefore, a substance such as Se that can interfere with ROS production could modulate SP signaling pathway to induce analgesia. Beta-endorphin (βE) produces analgesic effect by inhibiting the release of tachykinin (particularly SP) and GABA in the peripheral and central nervous system respectively which potentiate release of dopamine (Sprouse-Blum et al., 2010). The modulatory effects of Se on selected pain bio-molecular markers (Glutamate, SP, and βE) and pro-inflammatory cytokines (IL-1β, IL-6, and TNFα) in relation to pain perception was investigated in the MPTP-induced neurotoxicity.

## Materials and methods

### Chemicals

MPTP and DOPA were procured from MedChemExpress LLC (MedChemExpress, Princeton, NJ, USA). Sodium selenite and other reagents of analytical grade were purchased from Sigma Aldrich (St. Louis, MO, USA).

### Animals, MPTP induction and treatment with DOPA and Selenium

Male Swiss mice, 8–9 weeks old and weighing 19–24 g each were used for this study. These mice were obtained from the Central Animal Facility of University of Ilorin and housed, under standard housing condition (22 °C, 55% humidity and 12:12 h daylight/darkness cycles) at the Animal Experiment Laboratory, Central Research Laboratory, University of Ilorin, Ilorin, Nigeria with free access to standard laboratory feed and tap water. These mice were acclimatized for one week before the injection of MPTP in mice as approved in the study protocol.

**Fig. 1:**
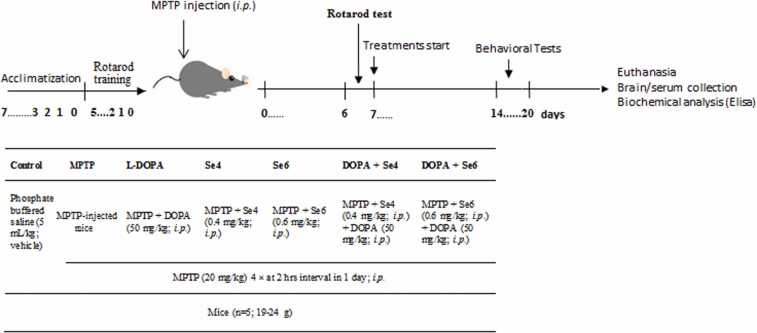
Experimental design showing the treatment time line. 1-methyl-4-phenyl-1,2,3,6-tetrahydropyridine; MPTP, intraperitoneal; *i.p.*, levodopa and benserazide combination; DOPA.

Three different sets of mice were used in order to reduce the number of painful experiments on the same animal. Each set contains 7 groups of 5 mice each. The first set of 35 mice was employed for motor function/coordination tests, the second set of mice was employed for thermal nociceptive tests and the third set of mice was employed for chemically-induced nociceptive tests. Neurotoxicity was induced *intraperitoneally* (*i.p*.) by injecting 30 mice with MPTP (20 mg/kg) dissolved in phosphate buffered saline (PBS) four times per day with an interval of 2 h between injections. Normal control mice were injected with an equal volume of PBS only. It has been established that approximately 70–90% loss of DA neurons is achieved 7 days after MPTP injection (Jackson-Lewis et al., 1995, Adeosun et al., 2012). Therefore, 7 days post MPTP injection, DOPA (50 mg/kg in PBS) was administered *i.p.* for 7 days to DOPA treated mice (DOPA), once daily. Similarly, Se at 0.4 mg/kg or 0.6 mg/kg in PBS was separately administered *i.p.* to different groups of MPTP-injected mice (Se4 and Se6). Also, combination of Se and DOPA was administered *i.p.* to other groups of MPTP-injected mice (DOPA+Se4 and DOPA+Se6) (Table 1). The selected doses of Se are within the range of doses previously reported (Ren et al., 2012, Jamba et al., 1997, Al-Harbi et al., 2014). Behavioral tests, commonly used to evaluate motor function/coordination (rotarod, grip, and open field test), mechanical nociception test (von Frey filament test), thermal nociception test (hot plate, hot water tail immersion, and cold water tail immersion tests), and chemical nociception test (formalin nociceptive tests) were conducted after seven days of DOPA/Se treatment in the mice (Fig. 1).

**Table 1:** Animal groupings and treatment.

S/N	Group	Description
1	Control	Phosphate buffered saline (0.2 ml/kg *i.p.*)
2	MPTP	MPTP (20 mg/kg *i.p.* four times at 2 h intervals in a day)
3	DOPA	MPTP + levodopa and benserazide combination (50 mg/kg *i.p.*)
4	Se4	MPTP + Se (0.4 mg/kg *i.p.*)
5	Se6	MPTP + Se (0.6 mg/kg *i.p.*)
6	DOPA + Se4	MPTP + DOPA + Se (0.4 mg/kg *i.p.*)
7	DOPA + Se6	MPTP + DOPA + Se (0.6 mg/kg *i.p.*)

MPTP, 1-methyl-4-phenyl-1,2,3,6-tetrahydropyridine. *i.p.*, intraperitoneal; (n = 5 mice per group).

### Behavioral tests

The behavioral tests were conducted 2 weeks post MPTP injection *i.e*. 7 days after treatment (Fig. 1). The experiment was performed in the standard laboratory conditions between 09.00 and 15.00 h. Observations and measurements were recorded by supporting staff that were blinded to the experimental design.

### Motor function/coordination tests

#### Rotarod test

The rotarod training was performed by maintaining the same duration of 10 min during five consecutive days: Mice were put on the rotarod at 4 rpm for 5 min on day one. Every 30 s, the rod speed was increased by 1 rpm up to 15 rpm, and then maintained this speed for one minute. A 4 rpm speed was used for 1.5 min on day two. The speed of the rotarod was increased by 1 rpm every 30 s until it reached 20 rpm, which was maintained for 10 min. On the third, fourth, and fifth day, the latencies of falling off rotarod were measured three times with linear increase of rotarod’s speed from 4 rpm up to 40 rpm for 5 min and averaged. On the sixth and seventh day of MPTP injection, the latencies of falling off rotarod were also measured three times with linear increase of rotarod’s speed from 4 rpm up to 40 rpm for 5 min. This was done to confirm motor decline. The actual test was performed after the drug administration (7th day of DOPA/Se treatment). The test protocol was the same with the last three days of the training protocol.

#### Grip test

A 55 cm wide and 2 mm thick wire was secured to two vertical stands and mice were subjected to hanging test by gently placing them at the central point of the wire and allowed to grip the wire with the forelimbs. The experiment was video covered to record the falling latency score of the mice.

#### Open field test

Each animal was placed in an open field maze (40 cm × 40 cm x 40 cm) made of white acrylic and video covered from above for 5 min. The number of lines crossed, total distance covered, speed, rearing, freezing frequency and freezing duration were evaluated in the open field test (OFT).

### Mechanical nociceptive tests

#### von Frey filament test

Each mouse was observed for the display of paw withdrawal reflex when the paw was unconsciously touched with von Frey filament. These filaments were applied to the plantar surface of the paw after the mouse is steady and in a comfortable position within the confined area with a perforated floor. The filaments were regulated to bend when the set force is applied to the paw. Filaments with various force rankings were applied in increasing order and animals were observed for provocation of response. The filament that initiated a response was identified and changed to the succeeding lower-rated filament. Conversely, when there was no response, the following higher filament was used, to ensure precise identification of minimal required force that produces a response. The experiment was repeated 4 times after the initial change in the animal’s position (Dixon, 1965).

### Thermal nociceptive tests

#### Hot plate test

The thermal nociceptive threshold was measured by putting mice on a hot plate having a constant temperature of 50 °C. The latency of flinching, licking, or jumping behavior was recorded. A cut-off time of 20 s was set to prevent tissue damage at 50 °C.

#### Hot water tail immersion test

Tail flick response was observed in each mouse when the tail is dipped in a water bath maintained at constant temperature (53 °C). The experiment was video covered and the time of reaction (tail flick) of the animal was recorded. A cut-off time of 15 s was set to prevent tissue damage at 53 °C.

#### Cold water tail immersion test

Tail flick response was observed in each mouse when the tail is dipped in a water bath maintained at constant temperature (4 °C). The experiment was video covered and the time of reaction (tail flick) of the animal was recorded. A cut-off time 30 s was set for cold water tail immersion test.

### Chemical nociceptive tests

#### Formalin nociceptive tests

Chemical pain was induced in mice by intraplantar injection of formalin, a chemical that acts on substance-P containing nociceptor. Mice were injected 0.2 ml of 5% formalin solution into the dorsal surface of the paw and returned into their cages. Video recording of the experiment started immediately after the injection of formalin into the paw to mark the beginning of phase 1 (acute phase). Paw-licking behavior was recorded for duration of 0–5 min. Then, licking time from 20 to 40 min after formalin injection was also recorded for phase II (tonic phase).

### Tissue sample collection and preparation

The mice were anesthetized using ketamine injection and blood samples were collected by cardiac puncture. Serum was separated by centrifugation for 10 min at 4 000 rpm and 4 °C. The resulting sera were aliquoted into Eppendorf tubes and kept at −20 °C until they were analyzed. The brains were immediately removed and kept on ice. The coronal section (1 mm) of the midbrain which contains the SNpc was dissected and the SNpc was separated from the adjacent ventral tegmental area as previously described (Lautenschläger et al. 2018) and guided by the mouse brain atlas (Paxinos and Franklin, 2004). The SNpc tissue samples were weighed and homogenized in 10 mg tissue/ml of cold lysis buffer (0.25 M sucrose and 1 tablet of complete mini protease inhibitor cocktail tablets/10 ml). Homogenates were centrifuged at 10,000×*g* for 40 min. The supernatant was collected into Eppendorf tubes and kept at −70 °C until they were analyzed.

### **Estimations of substantia nigra and serum levels of IL-1β, IL-6 and TNFα**

The SNpc and the serum concentrations of IL-1β, IL-6, and TNFα were measured using the ELISA method with monoclonal antibodies specific for mouse IL-1β, IL-6, and TNFα. Samples were run undiluted in duplicate and incubated on pre-coated plates. The optical density of each well was measured in accordance with the manufacturer’s instructions (Thermo-Fisher, Waltham, MA). The concentrations of IL-1β, IL-6, and TNFα were calculated against the standard curve and expressed as picogram per gram tissue or nanogram per gram tissue as the case may be.

### **Estimations of tyrosine hydroxylase, substance P, glutamate and β-endorphin in the substantia nigra**

The SNpc levels of SP, Glutamate, and βE were measured using the ELISA method with monoclonal antibodies specific for mouse TH, SP, Glutamate and βE. Samples were run undiluted in duplicate and incubated on pre-coated plates. The optical density of each well was measured using a microplate reader that was set to the corresponding wavelength as suggested by the manufacturer (Thermo-Fisher, Waltham, MA). The concentrations of SP, Glutamate, and βE were calculated against the standard curve and expressed as nanogram per gram of tissue, micromole per gram of tissue and microgram per gram of tissue, respectively.

### Data analysis

All data were presented as graphs (mean ± SD) and statistically analyzed using the GraphPad Prism (version 7.0, GraphPad Software Inc., San Diego, CA). Mice groups were compared using multifactorial ANOVA and Tukey’s *post-hoc* test was performed for multiple comparisons. a, b, c, d, e, and f represent statistically significant difference when compared to control, MPTP, DOPA, Se4, Se6, and DOPA+Se4 groups respectively at p < 0.05 except where otherwise stated.

## Results

### **Se or DOPA+Se partially restored motor deficits in MPTP-injected mice**

**Fig. 2:**
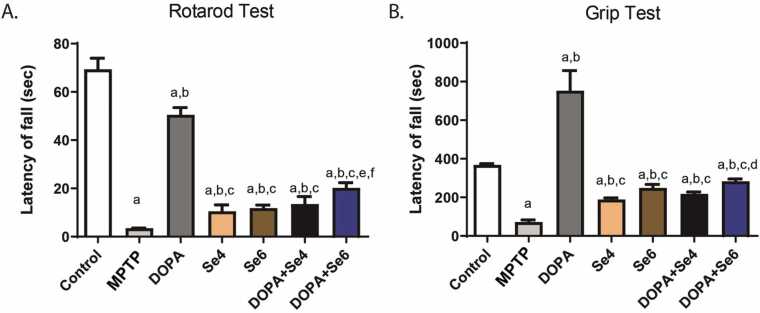
Locomotor activity of MPTP-injected mice treated with DOPA and Selenium. Latency of fall on Rotarod test (A), Latency of fall during Grip test (B), MPTP; MPTP lesioned mice, DOPA; DOPA (40 mg/kg)-treated MPTP-injected mice, Se4; Selenium (0.4 mg/kg)-treated MPTP-injected mice, Se6; Selenium (0.6 mg/kg)-treated MPTP-injected mice, DOPA+Se4; Selenium (0.4 mg/kg) and DOPA treated MPTP-injected mice, DOPA+Se6; Selenium (0.6 mg/kg) and DOPA treated MPTP-injected mice. Values are mean ± SD (n = 5). a, b, c, d, e, and f represent statistically significant difference when compared to control, PD, DOPA, Se4, Se6, and DOPA+Se4 groups respectively.

Motor activity, as evaluated by the rotarod test (Fig. 2A), was significantly (p < 0.0001) reduced in MPTP-injected mice as compared with the control or DOPA-treated group (3.53 ± 0.06 s *vs.* 69.34 ± 4.61 or 50.47 ± 3.06 s respectively). Treatment with Se (0.6 or 0.4 mg/kg) alone increased (p < 0.05) slightly spontaneous motor activity when compared with MPTP-injected mice (10.48 ± 2.71 or 11.81 ± 1.28 s *vs.* 3.53 ± 0.06 s respectively). Similarly, combination of Se (0.6 or 0.4 mg/kg) and DOPA increased spontaneous motor activity (13.50 ± 3.11 or 20.20 ± 2.18 s respectively; p < 0.001) when compared with MPTP-injected mice (3.53 ± 0.06 s). Distinctively, DOPA-treated mice exhibited a significant (p < 0.0001) increased spontaneous motor activity when compared with Se (0.6 or 0.4 mg/kg)-treated mice (50.47 ± 3.06 s *vs.* 10.48 ± 2.71 or 11.81 ± 1.28 s respectively) or DOPA+Se (0.6 or 0.4 mg/kg) treated MPTP-injected mice (50.47 ± 3.06 s *vs.* 13.50 ± 3.11 or 20.20 ± 2.18 s respectively) (Fig. 2A). Notably, Se (0.6 or 0.4 mg/kg), administered alone or in combination with DOPA (50 mg/kg), similarly produced partial recovery of motor deficits which was far below the effect of DOPA alone.

Moreover, in the grip test (Fig. 2B), there was a significant (p < 0.0001) reduction in muscle coordination in MPTP-injected mice when compared with the control or DOPA groups (71.69 ± 11.10 s *vs.* 368.09 ± 6.74 or 753.28 ± 103.57 s respectively). Se (0.6 or 0.4 mg/kg) increased muscle coordination significantly (p < 0.001) when compared with MPTP-injected mice (188.75 ± 8.54 or 248.50 ± 18.72 s *vs.* 71.69 ± 11.10 s respectively). However, treatment with DOPA alone increased significantly (p < 0.0001) muscle coordination in MPTP-injected mice as compared to control (753.28 ± 103.57 s *vs.* 368.09 ± 6.74 s respectively). Similarly, DOPA significantly (p < 0.0001) increased muscle coordination when compared with Se (0.6 or 0.4 mg/kg)-treated MPTP-injected mice (753.28 ± 103.57 s *vs.* 248.50 ± 18.72 or 188.75 ± 8.54 s respectively). By contrast, the level of muscle coordination observed, after co-administration of Se (0.6 or 0.4 mg/kg) and DOPA, was significantly (p < 0.001) lower as compared with DOPA alone (217.75 ± 10.01 or 283.25 ± 12.04 s *vs.* 753.28 ± 103.57 s respectively) but insignificantly different from treatment with Se only (Fig. 2B). This points to the fact that co-administration of Se and DOPA dampens the efficacy of administering DOPA alone in restoring motor activity and muscle coordination in the MPTP-injected mice. While administering Se alone improves motor activity and coordination, it proved not to be as effective as DOPA.

**Fig. 3:**
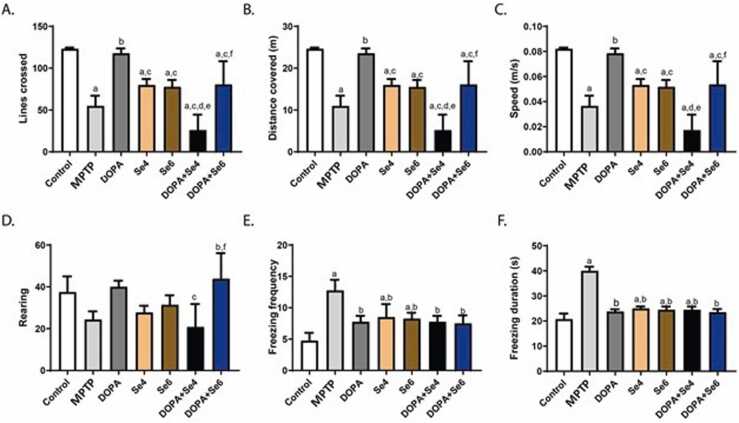
Locomotor activity of MPTP-injected mice treated with DOPA and Selenium. Exploratory motor performance, lines crossed (A), total distance covered (B), speed (C), rearing activity (D), freezing frequency (E), and freezing duration (F) in open field test. MPTP; MPTP lesioned mice, DOPA; DOPA (40 mg/kg)-treated MPTP-injected mice, Se4; Selenium (0.4 mg/kg)-treated MPTP-injected mice, Se6; Selenium (0.6 mg/kg)-treated MPTP-injected mice, DOPA+Se4; Selenium (0.4 mg/kg) and DOPA treated MPTP-injected mice, DOPA+Se6; Selenium (0.6 mg/kg) and DOPA treated MPTP-injected mice. Values are mean ± SD (n = 5). a, b, c, d, e, and f represent statistically significant difference when compared to control, PD, DOPA, Se4, Se6, and DOPA+Se4 groups respectively.

The number of lines crossed by MPTP-injected mice in OFT (Fig. 3A) was significantly (p < 0.0001) decreased when compared with control or DOPA treated groups (54.88 ± 12.22 *vs.*123.22 ± 1.35 or 117.84 ± 5.69 respectively), suggestive of decreased locomotion and exploratory activity in the acute MPTP-injected mice. DOPA increased the number of lines crossed significantly when compared with Se (0.6 or 0.4 mg/kg) (117.84 ± 5.69 *vs.* 79.91 ± 7.20 or 77.63 ± 8.34 respectively; p < 0.05). Likewise, DOPA increased the number of lines crossed significantly (p < 0.01) when compared to treatment with combination of Se (0.6 mg/kg) and DOPA (DOPA+Se6) (117.84 ± 5.69 *vs.* 80.50 ± 27.84 respectively). Se (0.6 or 0.4 mg/kg) administered separately or in combination with DOPA (DOPA+Se6) insignificantly (p > 0.05) increased the number of lines crossed *vs.* MPTP-injected mice (Fig. 3A).

The total distance traveled in OFT by the MPTP lesioned mice was significantly decreased relative to control or DOPA treated mice (10.98 ± 2.45 *vs* 24.64 ± 0.27 or 23.57 ± 1.14 respectively; p < 0.0001). DOPA significantly (p < 0.01) increased the total distance moved relative to Se (0.6 or 0.4 mg/kg) (23.58 ± 11.38 *vs.* 15.98 ± 1.44 or 15.53 ± 1.67 respectively). Also, DOPA significantly (p < 0.01) increased the total distance moved compared with combination of Se (0.6 mg/kg) and DOPA (DOPA+Se6) (23.58 ± 11.38 *vs.* 16.10 ± 5.57). Se (0.6 or 0.4 mg/kg) administered separately or in combination with DOPA (DOPA+Se6 or DOPA+Se4) showed no significant alteration (p > 0.05) in total distance moved *vs.* MPTP-injected mice (Fig. 3B).

Furthermore, the maximum speed moved in OFT by the MPTP lesioned mice was significantly low compared to control or DOPA treated mice (0.04 ± 0.01 *vs* 0.08 ± 0.00 or 0.08 ± 0.00 respectively; p < 0.0001). DOPA group was observed to have significantly (p < 0.01) increased movement speed compared to Se (0.6 or 0.4 mg/kg) (0.08 ± 0.00 *vs.* 0.05 ± 0.01 or 0.05 ± 0.01 respectively). Also, DOPA significantly (p < 0.01) increased the movement speed compared with combination of Se (0.6 mg/kg) and DOPA (DOPA+Se6) (0.08 ± 0.00 *vs.* 0.05 ± 0.02). Se (0.6 or 0.4 mg/kg) administered separately or in combination with DOPA (DOPA+Se6 or DOPA+Se4) showed no significant alterations (p > 0.05) in speed of movement *vs.* MPTP-injected mice (Fig. 3C). These results are suggestive of decreased locomotion and exploratory activity in the acute MPTP-induced neurotoxicity.

The rearing activity (Fig. 3D) was reduced albeit not significantly in MPTP-injected mice as compared with the control (normal) mice (24.38 ± 3.90 *vs.* 37.50 ± 7.55 respectively; p > 0.05). Treatment with DOPA in combination with Se (DOPA+Se6) significantly (p < 0.01) increased rearing activity when compared with MPTP-injected mice (43.88 ± 12.26 *vs.* 24.38 ± 3.90) (Fig. 3D).

There was a significant increase in the freezing frequency of MPTP lesioned mice compared to the freezing frequency of control and DOPA treated mice (12.75 ± 1.71 *vs* 4.75 ± 1.26 and 7.75 ± 0.96; p < 0.0001 respectively). There was no significant change in the freezing frequency of DOPA treated mice relative to Se (0.6 or 0.4 mg/kg) administered separately or in combination with DOPA *i.e*. DOPA+Se6 or DOPA+Se4. However, there was a significant (p < 0.01) decrease in the freezing frequency of Se4 (8.50 ± 2.08), Se6 (8.50 ± 0.96), DOPA+Se4 (7.75 ± 0.96) and DOPA+Se6 (7.50 ± 1.29) compared to the MPTP lesioned mice (12.75 ± 1.71) (Fig. 3E). Similarly, freezing duration of MPTP lesioned mice (40.0 ± 1.63) increased significantly (p < 0.0001) when compared to freezing duration of control (20.75 ± 2.22) and DOPA treated mice (23.75 ± 0.96). Nevertheless, Se4 (25 ± 0.82), Se6 (24.50 ± 1.29), DOPA+Se4 (24.50 ± 1.29) and DOPA+Se6 (23.50 ± 1.29) reduced freezing duration significantly (p < 0.01) compared to the MPTP lesioned mice (40.0 ± 1.63) (Fig. 3F). Se4, Se6 and DOPA+Se4 slightly increased freezing duration significantly (p < 0.01) compared to the control mice.

Taken together, these results show that the acute administration of MPTP produced motor deficits, a behavioral manifestation of MPTP-induced Parkinson-like disease. Administration of Se alone or combined with DOPA promotes partial motor function recovery significantly in some of the tests while DOPA alone restored motor function in most of the paradigms employed. Surprisingly, DOPA+Se produced no additive effects for motor function recovery.

### **Se or DOPA+Se increased the basal threshold of nociceptive stimuli in MPTP-injected mice**

**Fig. 4:**
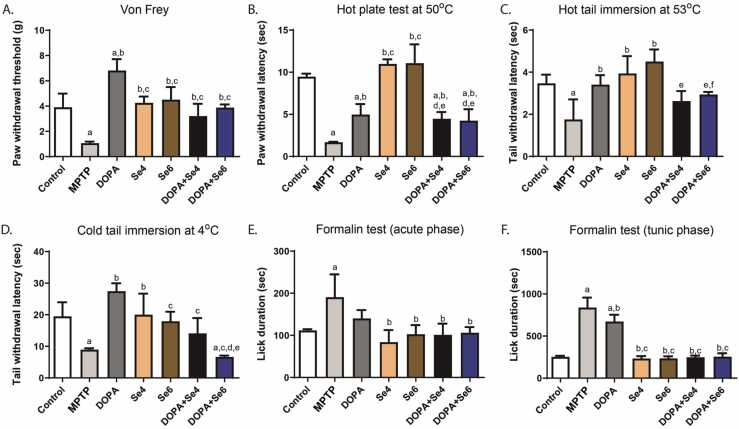
Baseline threshold of nociceptive sensitivity to mechanical, thermal, and chemical stimulation in MPTP-injected mice treated with DOPA and Se. Hind paw withdrawal latency (A), Thermal nociceptive threshold to constant thermal stimulation in hot plate test 50 °C (B), Nociceptive threshold in hot tail immersion at 53 °C (C), Nociceptive threshold in cold tail immersion test at 4 °C (D), Paw licking duration in the acute phase of formalin nociceptive tests (E), Paw licking duration tunic phase of formalin nociceptive tests (F). MPTP; MPTP lesioned mice, DOPA; DOPA (40 mg/kg)-treated MPTP-injected mice, Se4; Selenium (0.4 mg/kg)-treated MPTP-injected mice, Se6; Selenium (0.6 mg/kg)-treated MPTP-injected mice, DOPA+Se4; Selenium (0.4 mg/kg) and DOPA treated MPTP-injected mice, DOPA+Se6; Selenium (0.6 mg/kg) and DOPA treated MPTP-injected mice. Values are mean ± SD (n = 5). a, b, c, d, e, and f represent statistically significant difference when compared to control, PD, DOPA, Se4, Se6, and DOPA+Se4 groups respectively.

The threshold of basal nociceptive reaction to mechanical stimulation (von Frey stimulation) was significantly reduced in the MPTP-injected mice *vs.* the control and DOPA-treated (1.06 ± 0.13 g *vs.* 3.90 ± 1.09 g; p < 0.01 & 6.81 ± 0.90 g; p < 0.0001 respectively) MPTP-injected mice (Fig. 4A). Se (0.6 or 0.4 mg/kg) increased significantly (p < 0.001) the paw withdrawal threshold (4.25 ± 0.50 or 4.50 ± 1.00 g respectively) when compared with MPTP-injected mice (1.06 ± 0.13 g), and was comparable to the control mice (3.90 ± 1.09 g). Although, this effect of Se was lower significantly (p < 0.05) compared to DOPA-treated MPTP-injected mice (6.81 ± 0.90 g). In the same vein, combination of Se (0.6 or 0.4 mg/kg) and DOPA increased significantly (p < 0.05) the paw withdrawal threshold (3.20 ± 0.98 or 3.88 ± 0.25 g respectively) in relation to MPTP-injected mice (1.06 ± 0.13 g), although the effect was significantly (p < 0.001) lower when compared to DOPA treated MPTP-injected mice (Fig. 4A).

The threshold of basal nociceptive response to thermal stimulation on the 50 °C hotplate was significantly decreased in MPTP-injected mice when compared to control (p < 0.0001) and DOPA-treated MPTP-injected mice (p < 0.001) (1.68 ± 0.07 s *vs.* 9.47 ± 0.36 & 4.98 ± 1.25 s respectively). Se (0.6 or 0.4 mg/kg)-treated MPTP-injected mice showed a significant (p < 0.0001) increase in the paw withdrawal latency (10.98 ± 0.55 or 11.08 ± 2.23 s respectively) when compared to the MPTP-injected mice (1.68 ± 0.07 s) and the DOPA-treated MPTP-injected mice (4.98 ± 1.25 s). Co-administration of Se (0.6 or 0.4 mg/kg) and DOPA significantly (p < 0.01) increased the thermal nociceptive response *vs.* parkinsonian MPTP-injected mice (4.47 ± 0.79 or 4. 24 ± 1.36 s *vs.* 1.68 ± 0.07 s respectively) and the effect was similar to that observed in DOPA alone (4.98 ± 1.25 s) (Fig. 4B).

Consistently, Se (0.6 or 0.4 mg/kg)-treated mice presented with significant (p < 0.001) increase in the tail withdrawal latency in relation to the MPTP-injected mice (3.94 ± 0.83 or 4.50 ± 0.58 s *vs.* 1.75 ± 0.96 s respectively) in hot tail immersion at 53 °C, and this effect was similar to the control (3.47 ± 0.41 s) and the DOPA-treated mice (3.41 ± 0.45 s). Surprisingly, Co-administration of Se (0.6 or 0.4 mg/kg) and DOPA showed no significant (p > 0.05) alteration in the tail withdrawal latency relative to the MPTP-injected mice (Fig. 3C). In addition, the tail withdrawal latency of the MPTP-injected mice to cold stimulation was significantly reduced in relation to control (p < 0.05) and DOPA (P < 0.0001) (8.93 ± 0.45 s *vs.* 19.48 ± 4.49 & 27.46 ± 2.49 s respectively). Treatment with Se (0.6 or 0.4 mg/kg) increased significantly (p < 0.05) the tail withdrawal latency relative to the MPTP-injected mice (20.03 ± 6.64 or 17.91 ± 3.03 s *vs.* 8.93 ± 0.45 s respectively) and this result was similar to the control (19.48 ± 4.49 s) and DOPA-treated MPTP-injected mice (27.46 ± 2.49 s). Concomitant administration of Se and DOPA showed no significant alteration in the tail withdrawal latency in relation to the MPTP-injected mice (Fig. 4D).

Moreover, following formalin injection into the hind-paw, MPTP-injected mice immediately (0.2 s) started licking and biting the hind-paw vigorously and continued throughout the 5 min test period (acute phase). Strikingly, Se (0.6 or 0.4 mg/kg)-treated MPTP-injected mice showed a significantly (p < 0.01) reduced paw licking duration (83.75 ± 28.76 or 102.50 ± 21.81 s respectively) relative to the MPTP-injected mice (190.50 ± 54.49 s). Likewise, co-administration of Se (0.6 or 0.4 mg/kg) and DOPA decreased licking duration (101.00 ± 26.77 or 106.00 ± 13.66 s respectively) in the manner similar to Se alone while no significant change was observed in mice treated with DOPA alone (140.00 ± 20.07 s) when compared to MPTP-injected mice (190.50 ± 54.49 s) (Fig. 4E). The duration of the nociceptive response displayed by MPTP-injected mice was significantly (p < 0.0001) increased in the tonic phase when compared to control (837.75 ± 118.97 *vs.* 253.50 ± 12.61 s respectively). However, treatment with Se (0.6 or 0.4 mg/kg) reduced significantly (p < 0.0001) the licking duration (233.00 ± 29.31 or 234.75 ± 24.72 s respectively) when compared with MPTP-injected mice (837.75 ± 118.97 s) and the DOPA-treated MPTP-injected mice (671.25 ± 82.30 s). Similarly, combination of Se (0.6 or 0.4 mg/kg) and DOPA significantly (p < 0.0001) reduced the licking duration (247.75 ± 20.66 or 254.50 ± 41.35 s respectively) when compared with MPTP-injected mice (837.75 ± 118.97 s) and the DOPA-treated MPTP-injected mice (671.25 ± 82.30 s) (Fig. 4F). Notably, DOPA-treated MPTP-injected mice exhibited no significant (p > 0.05) change in the nociceptive response duration when compared to MPTP-injected mice at the acute and tonic phases of the formalin test.

### **Se reduced substantia nigra and serum pro-inflammatory cytokines levels in MPTP-injected mice**

**Fig. 5:**
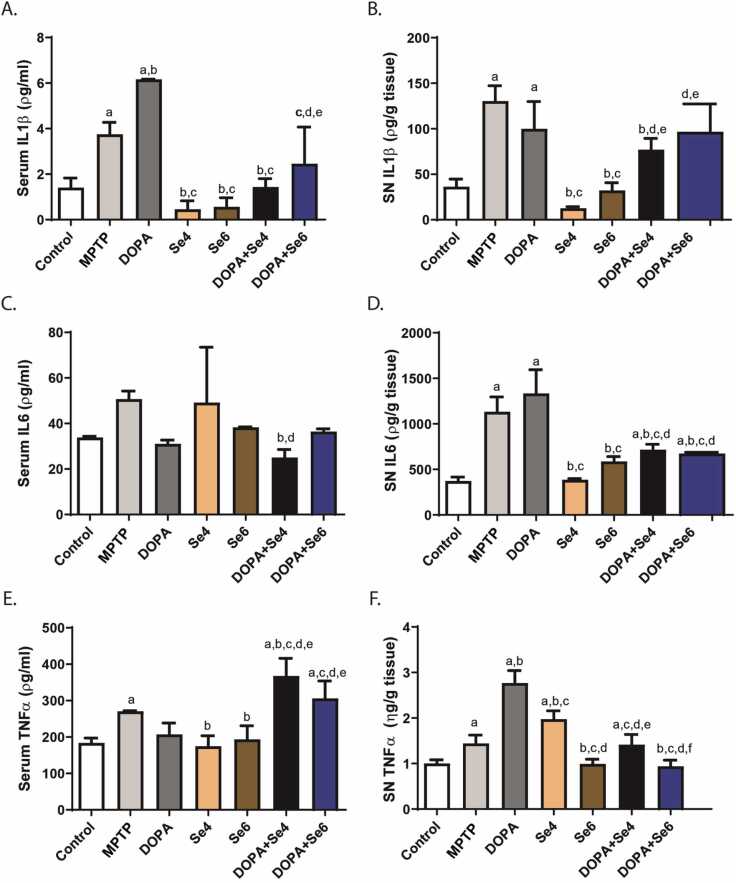
Serum and substantia nigra pars compacta (SNpc) pro-inflammatory cytokines levels in MPTP-injected mice treated with DOPA and Se. Serum level of IL-1β (A), SNpc level of IL-1β (B), Serum level of IL-6 (C), SNpc level of IL-6 (D), Serum level of TNFα (E), SNpc level of TNFα (F). MPTP; MPTP lesioned mice, DOPA; DOPA (40 mg/kg)-treated MPTP-injected mice, Se4; Selenium (0.4 mg/kg)-treated MPTP-injected mice, Se6; Selenium (0.6 mg/kg)-treated MPTP-injected mice, DOPA+Se4; Selenium (0.4 mg/kg) and DOPA treated MPTP-injected mice, DOPA+Se6; Selenium (0.6 mg/kg) and DOPA treated MPTP-injected mice. Values are mean ± SD (n = 5). a, b, c, d, e, and f represent statistically significant difference when compared to control, PD, DOPA, Se4, Se6, and DOPA+Se4 groups respectively.

We evaluated systemic (serum) and SNpc levels of the proinflammatory cytokines: IL-1β, IL-6, and TNFα. A significant increase in the serum levels of IL-1β (3.75 ± 0.52 pg/ml; p < 0.01), IL-6 (50.67 ± 3.55 pg/ml; p < 0.01) and TNFα (270.69 ± 1.49 pg/ml; p < 0.05) was observed in the MPTP-injected mice when compared with serum levels of IL-1β (1.41 ± 0.42 pg/ml), IL-6 (33.86 ± 0.48 pg/ml) and TNFα (184.09 ± 13.38 pg/ml) respectively in control mice (Fig. 5A,C,E). Treatment of MPTP-injected mice with Se (0.6 or 0.4 mg/kg) alone significantly decreased the levels of serum IL-1β (0.46 ± 0.37 or 0.57 ± 0.40 pg/ml respectively; p < 0.0001) and TNFα (174.88 ± 28.71 or 193.70 ± 37.52 pg/ml respectively; p < 0.01) without a significant change in IL-6 (49.19 ± 24.30 or 38.28 ± 0.23 pg/ml; p > 0.05) while treatment with DOPA further elevated serum level of IL-1β (6.17 ± 0.00 pg/ml; p < 0.01) and decreased serum IL-6 (31.03 ± 1.67 pg/ml; p < 0.01) without a significant change in the serum level of TNFα (207.44 ± 31.06 pg/ml; p > 0.05) when compared with the serum levels of IL-1β (1.41 ± 0.42 pg/ml), IL-6 (50.67 ± 3.55 pg/ml) and TNFα (270.69 ± 1.49 pg/ml) in the MPTP-injected mice (Fig. 5A,C,E). Co-administration of Se (0.6 or 0.4 mg/kg) and DOPA reduced significantly serum IL-1β (1.43 ± 0.37 or 2.46 ± 1.61 pg/ml respectively; p < 0.0001) and IL-6 (25.05 ± 3.50 or 36.47 ± 1.17 pg/ml respectively; p < 0.05) levels when compared with serum IL-1β (3.75 ± 0.52 pg/ml) and IL-6 (50.67 ± 3.55 pg/ml) respectively in MPTP-injected mice. However, this combination (DOPA+Se4 or DOPA+Se6) elevated TNFα level (367.61 ± 48.62 or 306.12 ± 47.80 pg/ml respectively) significantly (p < 0.01) when compared with the MPTP-injected mice serum TNFα level (270.69 ± 1.49 pg/ml) (Fig. 5A,C,E).

In the SNpc, IL-1β, IL-6, and TNFα levels were significantly (130.41 ± 16.85 pg/g; p < 0.0001, 1132.40 ± 162.95 pg/g; p < 0.0001 and 1.44 ± 0.19 ng/g; p < 0.05 respectively) elevated in MPTP-injected mice relative to the SNpc levels of IL-1β, IL-6, and TNFα in controls (36.41 ± 8.35 pg/g, 371.89 ± 44.11 pg/g, 1.00 ± 0.08 ng/g respectively) (Fig. 5B,D,F). Treatment with DOPA alone had no significant effect on SNpc levels of IL-1β and IL-6 (Fig. 5B,F) but elevated the level of TNFα significantly (2.77 ± 0.27 ng/g; p < 0.0001) when compared to MPTP-injected mice (1.44 ± 0.19 ng/g) (Fig. 5F). Unlike DOPA, treatment with Se (0.6 or 0.4 mg/kg) significantly decreased SNpc levels of IL-1β (12.73 ± 1.75 or 32.29 ± 8.43 pg/g respectively; p < 0.0001), IL-6 (386.36 ± 12.68 or 586.69 ± 54.32 pg/g respectively; p < 0.0001), and TNFα (1.97 ± 0.19 or 0.99 ± 0.10 ng/g respectively; p < 0.05) relative to the SNpc levels of IL-1β (130.41 ± 16.85 pg/g), IL-6 (1132.40 ± 162.95 pg/g), and TNFα (1.44 ± 0.19 ng/g) respectively in the MPTP-injected mice (Fig. 5B,D,F). The beneficial effect of Se to decrease SNpc levels of the proinflammatory cytokines was not lost by its combination with DOPA, however, the proinflammatory cytokine-suppressive effect of Se was weakened by the combination significantly in the case of IL-1β (Fig. 5B).

Taken together, these results show that DOPA exacerbates or has no effect on inflammation characteristic of PD while Se proves to be a potent anti-inflammatory agent. The combination of Se and DOPA does not provide any extra benefit compared to administering Se alone.

### **Se or DOPA+Se increased tyrosine hydroxylase similar to control in the substantia nigra of MPTP-injected mice**

**Fig. 6:**
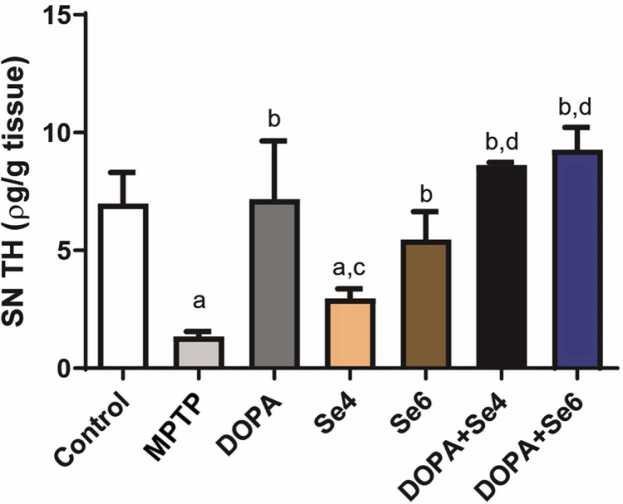
Tyrosine hydroxylase concentration in substantia nigra pars compacta (SNpc) of MPTP-injected mice treated with DOPA and Se. MPTP; MPTP lesioned mice, DOPA; DOPA (40 mg/kg)-treated MPTP-injected mice, Se4; Selenium (0.4 mg/kg)-treated MPTP-injected mice, Se6; Selenium (0.6 mg/kg)-treated MPTP-injected mice, DOPA+Se4; Selenium (0.4 mg/kg) and DOPA treated MPTP-injected mice, DOPA+Se6; Selenium (0.6 mg/kg) and DOPA treated MPTP-injected mice. Values are mean ± SD (n = 5). a, b, c, d, e, and f represent statistically significant difference when compared to control, PD, DOPA, Se4, Se6, and DOPA+Se4 groups respectively.

TH deficiency is a hallmark in MPTP-induced Parkinson-like disease. As expected, MPTP depleted significantly (p < 0.0001) TH levels in the SNpc of MPTP-injected mice when compared with control and DOPA groups (1.35 ± 0.21 pg/g *vs.* 6.98 ± 1.33 & 7.17 ± 2.48 pg/g respectively) (Fig. 6). Treatment with DOPA (p < 0.0001) or Se (0.6 mg/kg; p < 0.01) increased TH levels similarly (7.17 ± 2.48 or 5.45 ± 1.19 pg/g respectively) and were comparable to control (6.98 ± 1.33 pg/g) level when compared with MPTP-injected mice (1.35 ± 0.21 pg/g). An attempt to evaluate the combined (potential additive) effect of DOPA and Se revealed that administration of DOPA + Se (0.6 or 0.4 mg/kg) increased TH level when compared with MPTP-injected mice (8.62 ± 0.11 or 9.27 ± 0.96 pg/g *vs.* 1.35 ± 0.21 pg/g respectively; p < 0.0001) (Fig. 6). While this combination increased TH levels better than Se alone, it, however, does not provide extra benefit when compared with mice that received DOPA only. Thus, the combinatorial effect of administering DOPA + Se to MPTP-injected mice is probably driven mostly by DOPA.

### **Se or DOPA+Se reduced substance P, glutamate, without a change in β-endorphin level in the substantia nigra of MPTP-injected mice**

**Fig. 7:**
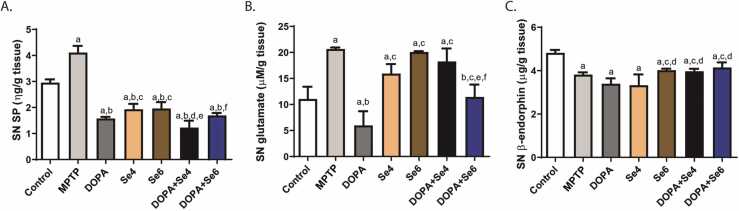
Substantia nigra pars compacta (SNpc) concentration of pain biomarkers in MPTP-injected mice treated with DOPA and Se. SNpc concentration of substance P (A), SNpc concentration of glutamate (B), SNpc concentration of β-endorphin (C). MPTP; MPTP lesioned mice, DOPA; DOPA (40 mg/kg)-treated MPTP-injected mice, Se4; Selenium (0.4 mg/kg)-treated MPTP-injected mice, Se6; Selenium (0.6 mg/kg)-treated MPTP-injected mice, DOPA+Se4; Selenium (0.4 mg/kg) and DOPA treated MPTP-injected mice, DOPA+Se6; Selenium (0.6 mg/kg) and DOPA treated MPTP-injected mice. Values are mean ± SD (n = 5). a, b, c, d, e, and f represent statistically significant difference when compared to control, PD, DOPA, Se4, Se6, and DOPA+Se4 groups respectively.

The SNpc level of SP increased significantly (p < 0.0001) in MPTP-injected mice when compared with control (4.11 ± 0.26 ng/g *vs.* 2.96 ± 0.13 ng/g respectively). However, Se (0.6 or 0.4 mg/kg) reduced (p < 0.0001) the SNpc level of SP in relation to MPTP-injected mice (1.93 ± 0.21 or 1.96 ± 0.24 *vs.* 4.11 ± 0.26 ng/g respectively). Similarly, combination of Se (0.6 or 0.4 mg/kg) and DOPA (50 mg/kg) significantly (p < 0.0001) reduced the SNpc level of SP in relation to control and MPTP-injected mice (1.23 ± 0.27 or 1.69 ± 0.09 *vs.* 2.96 ± 0.13 ng/g & 4.11 ± 0.26 ng/g respectively) and this was similar to DOPA (1.58 ± 0.06 ng/g) (Fig. 7A). MPTP-injected mice had a significant (p < 0.0001) increase in the SNpc glutamate concentration compared to the control (20.67 ± 0.28 µM/g *vs.* 11.05 ± 2.35 µM/g). Treatment with DOPA significantly (p < 0.0001) decreased the SNpc glutamate concentration relative to control or MPTP-injected mice (5.98 ± 2.72 *vs.* 11.05 ± 2.35 or 20.67 ± 0.28 µM/g respectively). However, treatment with Se alone had no significant effect on glutamate levels compared to the MPTP-injected mice. The combination of Se (0.6 mg/kg) and DOPA (DOPA+Se6) significantly decreased glutamate level relative to MPTP-injected mice (11.46 ± 2.36 µM/g *vs.* 20.67 ± 0.28 µM/g). However, this combination was not as effective as DOPA alone (Fig. 7B). MPTP-injected mice exhibited significant (p < 0.001) decrease in the SNpc level of β-endorphin (βE) compared to the control (3.82 ± 0.11 µg/g *vs.* 4.82 ± 0.14 µg/g). Likewise, treatment with Se and DOPA, combined or separately, showed no significant (p > 0.05) alterations in the SNpc level of βE when compared with MPTP-injected mice (Fig. 5C).

## Discussion

Acute *intraperitoneal* injection of MPTP prompted changes in the behavioral reactions to mechanical, thermal, and chemical pain stimulations. Similarly, there was manifestation of poor motor functions in the batteries of tests used to confirm motor deficits in the MPTP-injected mice model and this is consistent with previous reports (Sian et al., 1999, Park et al., 2015). However, we observed partial motor function recovery upon treatment with either Se only or combined with DOPA. Specifically, DOPA-treated MPTP-injected mice displayed a full motor function recovery to the control level. Se supplementation has been reported to inhibit the toxicity of DA neurons and protect neurons in the SNpc and nigrostriatal axis from damage triggered by oxidative stress in both MPTP- and 6-OHDA-induced PD model (Kim et al. 2000). In line with these, the results showed that injection of Se produced partial or sustained improvement in motor coordination and exploration activities albeit not as effective as DOPA. This suggests that the antioxidant effects of Se (Huang et al., 1994, Jiménez‐Jiménez et al., 1995) at maximal efficacy may have beneficial influences for partial improvement of motor deficit in MPTP-injected mice. Surprisingly, the combination of Se and DOPA lacks an additive effect on motor function recovery.

Generally, rodent models of PD exhibit increased sensitivity to pain as shown in the current and previous studies (Kim et al., 2000, Park et al., 2015). A lower nociceptive threshold is an important non-motor clinical symptom of PD. The dopaminergic system, in the SNpc, is often involved in pain perception. Inhibition or activation of dopamine D2 receptor is associated with nociceptive reactions (Magnusson and Fisher, 2000). A report from a human study also confirmed the role of dopamine D2 receptors in pain (Hagelberg et al. 2004). Moreover, MPTP-induced neurotoxicity decreased nociceptive threshold and administration of DOPA alone remarkably increased the basal nociceptive threshold of mechanical pain stimulation and cold tail immersion (4 °C). These results revealed that the dopaminergic axis has a role to play in inhibiting basal mechanical and thermal nociception. Although, SNpc dopaminergic axis may not be the main axis for modulating nociceptive thresholds of mechanical, thermal, or chemical stimuli, the spinal cord DA system also plays key roles in motor function as well as nociception. For instance, stimulation of dopamine D2 and D3 receptors in the spinal cord was shown to alter antinociception (Levant and McCarson, 2001) through descending inhibition (Gao and Mason, 2000). Although, DOPA increases nociceptive threshold in both thermal pain stimulations (50 °C or 53 °C) by increasing paw withdrawal or tail withdrawal latency respectively, the effect of Se alone is more beneficial in reducing the basal nociceptive sensitivity to thermal stimulations especially at a high temperature (53 °C) compared to treatment with DOPA or its combination with Se. Again, Se alone remarkably alleviated chemical nociception in both acute and tonic phases of chemical pain stimulation with no extra benefit offered by its combination with DOPA. Park et al. (2015) reported that DOPA increased response latency in mechanical stimulation but lacks antinociceptive effect following thermal stimulation at a high temperature which is consistent with the current findings. This shows that Se is a potential analgesic adjuvant for relieving PD-associated pain, especially where the pain is chemically or thermally provoked with little benefit for PD associated motor deficits.

There have been limitations in the body of knowledge defining pain relief management and the exact mechanism of hypersensitivity to pain in PD. Nevertheless, it has been demonstrated that progressive loss of DA neurons in SNpc provokes astrogliosis and microglial activation which are directly associated with neuroinflammation and dyshomeostasis in the basal ganglia and its circuitry progressions (Ha and Jankovic, 2012). Other reports have also shown that *intrastriatal* 6-OHDA induced a moderate increase in astrocyte activation in the basal ganglia and the dorsal horn of spinal cord (Lautenschläger et al. 2018). In this study, we showed a considerable increase in the serum and SNpc concentrations of pro-inflammatory cytokines (IL-1β, IL-6, and TNFα), suggesting systemic and remote local inflammation. Although the exact mechanism underlying the increase in the serum concentration of IL-1β, IL-6, and TNFα was not determined, it has been reported that ingestion of toxic agents like MPTP induces inflammation of hepatocytes, activation of Kupffer cells, and increase production of the peripheral cytokines (Henning et al., 2008, Barak et al., 2009, Loftis et al., 2008). It has been shown that peripheral inflammation is one of the critical factors that trigger exacerbation in central progression of brain damage in PD and its associated sickness behaviors (Ferrari and Tarelli, 2011). Therefore, the increased SNpc concentrations of pro-inflammatory cytokines that we observed in this study might have been through: migration of these cytokines from the serum into the brain through the neural pathway (the vagal afferents to the liver), breakdown in the integrity of blood-brain barrier (D'Mello and Swain, 2011), or astrocyte-derived pro-inflammatory cytokines. Hence, we posit hypothetically that the increased pro-inflammatory cytokines in the SNpc might have been due to any of those mechanisms mentioned. Additionally, microglia activation remotely increases neurotoxicity with consequential neuronal death through the release of free radicals and an amplified release of pro-inflammatory cytokines contributing to the progression of PD. Taken together, our result is correlating MPTP-induced neurotoxicity to hyper-release of pro-inflammatory cytokines which ultimately affects pain processing in the brain.

In light of pain mechanisms, increased pro-inflammatory cytokines have been associated with hyperalgesia through the stimulatory effects of inflammatory cytokines on the release of pain modulators, specifically glutamate and SP (Le and Adam, 2002). IL-1β, IL-6, and TNFα have been reported to play a facilitatory role in increasing glutamate production by increasing the activity of neuronal glutaminase enzyme (Clark and Vissel, 2016). Although the activity of glutaminase enzyme was not investigated in this study, elevated SNpc glutamate concentration that we observed might have been mediated through increased SNpc levels of pro-inflammatory cytokines. It has been previously shown that TNFα acts by inhibiting glial glutamate transporters on astrocytes and that SP stimulates the release of IL-6 in the central nervous system (Clark and Vissel, 2016). Again, IL-1β and IL-6 were reported to impair glutamate uptake by oxidative stress or NO (Le and Adam, 2002). These effects might, however, be paradoxical on the βE system which takes part in the control of the pain descending inhibitory system. It is well known that pro-inflammatory cytokines are elevated in mental fatigue and major depression. These might not be unconnected with inhibitory effects of pro-inflammatory cytokines on the βE system. Our study, therefore, supports this hypothesis since MPTP-injected mice were shown to display a low level of βE which correlates hyperalgesia to loss of descending pain inhibitory system. The mice were also shown to have reduced rearing activities and line crossing, the indices of motor dysfunction, and depression.

We also found that treatment with Se alone reduced both serum (IL-1β and IL-6) and SNpc (IL-1β, IL-6 and TNFα) levels of pro-inflammatory cytokines with no extra benefit offered by the combination of Se and DOPA. This may not be unconnected with the antioxidant effects of Se in the SNpc of parkinsonian mice previously reported (Zafar et al. 2003). It has been shown that Se, *via* its antioxidant property and interaction with Fe and Ca ion channels, enhanced activity of endogenous antioxidant defense enzymes particularly glutathione peroxidase. It prevented Fenton reaction and blocked Ca ion channels to halt dopamine depletion, neuronal membrane lipid peroxidation, and Ca homeostasis impairment. These ultimately inhibit neurodegeneration in the SNpc of a PD model (Zafar et al. 2003). Thus, neuronal oxidative assault which might trigger astrocyte and microglial activation to cause upregulation of pro-inflammatory cytokines is prevented by Se administration. Of note, the administration of DOPA alone exacerbates MPTP-induced neuroinflammation or had no effect in the serum and SNpc. The reason for this is still elusive. However, this increase might be physiological because pro-inflammatory cytokines might be protective in function considering the fact that the set of mice in this group displayed remarkable low pain sensitivity. Further studies are required to understand the exact mechanism by which Se downregulated pro-inflammatory cytokines in the SNpc of the MPTP-injected mice.

In the current study, MPTP-injected mice exhibits decreased nociceptive threshold, and increased pro-inflammatory cytokines, SP, and glutamate concentrations in the SNpc while the concentration of βE was considerably low relative to controls. Put together, these results linked neurotoxicity due to the increased level of pro-inflammatory cytokines and pain biomarkers to hyperalgesia in MPTP-injected mice. However, treatment with Se alone or combination of Se and DOPA substantially decreased nociceptive response in the MPTP-injected mice, perhaps by down-regulation of pro-inflammatory cytokines and modulation of pain biomarkers in the SNpc of MPTP-injected mice. Though, administration of Se alone or combination of Se and DOPA showed no significant alterations in the SNpc levels of βE. Se produced it antinociception *via* modulation of SP while DOPA modulates both SP and glutamate levels in the SNpc of MPTP-injected mice. These might account for the differential anti-nociceptive effects of Se and DOPA in response to painful stimuli of different origins (mechanical, thermal, and chemical) and lack of additive effect when both agents were administered.

The extensive connection between the DA system and the basal ganglia suggests that PD-associated pain could have originated from neuroinflammation or neurotoxicity that resulted in dysfunctional basal ganglia, DA neurons, sensory pathways within the basal ganglia, and thalamocortical-basal ganglia circuits. Evidence for this is the reduced level of SNpc glutamate and the corresponding decrease in nociception in the DOPA-treated group particularly during mechanical pain stimulation. This goes in line with the effect of DOPA on musculoskeletal pain, where PD patients feel relief after DOPA therapy, and the increased pain threshold noted when the medication concentration is highest (Nebe and Ebersbach, 2009). Perhaps, regulation of neuroinflammation within the basal ganglia and its circuitry coupled with glutamate and SP modulation, mediate Se- or DOPA+Se-induced analgesia in the MPTP-induced neurotoxicity. There are few limitations in this study that could be addressed in future research. First, the sample size used in this study is relatively small. The sample size can influence the significant relationships observed in this study. Second, the Lautenschlager method adopted for the removal of SNpc is known to be less accurate for the delineation of SNpc and SNpr as both tissues are collected by this method. Thus, studies that would use higher sample size, especially for the behavioral studies, and modified or accurate method for SNpc removal, are required to establish the current findings.

## Conclusion

The hyper-responsiveness to nociceptive stimuli which is one of the most prominent non-motor symptoms of PD might be due to neuroinflammation signified by upregulation of evaluated pro-inflammatory cytokines in the serum and SNpc leading to dyshomeostasis of pain biomarkers in the SNpc of MPTP-injected mice. However, DOPA consistently improved motor coordination and general exploratory activity as they are related to endurance and anxiety respectively while Se alone or combination of Se and DOPA improved general exploratory activity with a limited positive effect on motor coordination and endurance. Se and DOPA increased nociceptive threshold appreciably but Se alone remarkably increased chemically and thermally evoked nociceptive threshold. Se might suppress painful stimuli *via* inhibition of neuroinflammation and neuronal oxidative assault (Zafar et al. 2003) in the SNpc to halt neuroinflammation-induced alteration of basal pain biomarkers (SP and glutamate) in the SNpc of MPTP-injected mice.

## Ethical statement

Animal handling and the experimental procedure were carried out in accordance with international guidelines for the care and use of laboratory animals. The study protocol was approved by the University of Ilorin Ethical Review Committee (UERC/ASN/2017/738).

## Author contributions

POA and ABN conceived the idea. POA, ABN and ALO designed the experiment and collected the data. AA, ALO and OMO participated in the research conceptualization, data analysis and manuscript drafting. BVO and ABN review the design of the experiment, edited the manuscript and supervised the work. All authors have read and approved the final manuscript.

## Funding

This work was supported by the 10.13039/501100008895Tertiary Education Trust Fund (TETFund) of the Federal Government of Nigeria under Grant TETFUND/DESS/NRF/STI/11/Vol.1.

## Conflicts of interest

The authors report no declarations of interest.
